# Unravelling the dynamics of mental health inequalities in England: A 12-year nationwide longitudinal spatial analysis of recorded depression prevalence

**DOI:** 10.1016/j.ssmph.2024.101669

**Published:** 2024-04-15

**Authors:** Dialechti Tsimpida, Anastasia Tsakiridi, Konstantinos Daras, Rhiannon Corcoran, Mark Gabbay

**Affiliations:** a Department of Public Health, Policy and Systems, University of Liverpool, UK; b Centre for Research on Ageing, University of Southampton, UK; c Department of Gerontology, University of Southampton, UK; d Geographic Information Systems (GIS) Independent Researcher, UK; e National Institute for Health Research Applied Research Collaboration North West Coast (NIHR ARC NWC), UK; f Department of Primary Care and Mental Health, University of Liverpool, UK

**Keywords:** Mental health, Inequalities, Spatial statistics, Depression, Morans' I, Geographically weighted regression

## Abstract

**Background:**

Depression is one of the most significant public health issues, but evidence of geographic patterns and trends of depression is limited. We aimed to examine the spatio-temporal patterns and trends of depression prevalence among adults in a nationwide longitudinal spatial study in England and evaluate the influence of neighbourhood socioeconomic deprivation in explaining patterns.

**Methods:**

Information on recorded depression prevalence was obtained from the indicator Quality and Outcomes Framework: Depression prevalence that measured the annual percentage of adults diagnosed with depression for Lower Super Output Areas (LSOA) from 2011 to 2022. We applied Cluster and Outlier Analysis using the Local Moran’s I algorithm. Local effects of deprivation on depression in 2020 examined with Geographically Weighted Regression (GWR). Inequalities in recorded prevalence were presented using Prevalence Rate Ratios (PRR).

**Results:**

The North West Region of England had the highest concentration of High-High clusters of depression, with 17.4% of the area having high values surrounded by high values in both space and time and the greatest percentage of areas with a high rate of increase (43.1%). Inequalities widened among areas with a high rate of increase in prevalence compared to those with a lower rate of increase, with the PRR increasing from 1.66 (99% CI 1.61–1.70) in 2011 to 1.81 (99% CI 1.76–1.85) by 2022. Deprivation explained 3%–39% of the variance in depression in 2020 across the country.

**Conclusions:**

It is crucial to monitor depression's spatial patterns and trends and investigate mechanisms of mental health inequalities. Our findings can help identify priority areas and target prevention and intervention strategies in England. Evaluating mental health interventions in different geographic contexts can provide valuable insights to policymakers on the most effective and context-sensitive strategies, enabling them to allocate resources towards preventing the worsening of mental health inequalities.

## Highlights

• The first nationwide, longitudinal spatial analysis of depression.
• Rise in depression rates from 2011 to 2022 throughout England, but the increase was not uniform.
• Mental health inequalities are widening more rapidly in some areas.
• Influence of neighbourhood socio-economic deprivation in shaping the spatial distribution of depression.
• Spatial modelling plays a crucial role in researching and preventing the exacerbation of mental health inequalities.

## 1 Introduction

Depression is a major public health issue that can significantly impact an individual’s quality of life (Herrman et al., 2019). It is one of the most common mental health disorders and a leading cause of disability worldwide. According to the most recent data from 2019, depression affects an estimated 280 million people globally, as reported by the World Health Organization in 2021 (WHO, 2021). Additionally, studies have indicated an increase of 53.2 million cases of depression worldwide due to the COVID-19 pandemic (Santomauro et al., 2021).

Prior research has primarily concentrated on the person-centred medical aspect of depression (Marwaha et al., 2022) and neglected to examine its geographical distribution and connections to underlying factors (Rivera & Mollalo, 2022); nevertheless, a growing body of research now reports the role of place-based factors in determining mental health (Corcoran et al., 2022; Graif, Arcaya, & Diez Roux, 2016; Rivera & Mollalo, 2022).

Furthermore, due to advancements in computing power and more recent spatio-temporal models, such as space-time patterns mining analytics (i.e., space-time emerging hot spots analysis), there are new possibilities regarding the analysis and modelling of spatial data (Lin & Wen, 2022; Naqvi et al., 2021). In their recent review, Smith-East and Neff (Smith-East & Neff, 2020) emphasised the importance of utilising spatial statistics and Geographical Information Systems (GIS) to identify factors contributing to the spatial variations of depression. The spatial statistics methodology acknowledges that space influences the observations and shows that nearby units are associated (Getis & Ord, 1992). This means that spatio-temporal epidemiological models can reveal patterns of depression between different places and demonstrate how these patterns change over time; trends that conventional statistics are not suitable to handle (Rivera & Mollalo, 2022).

Our scoping review showed that the few studies that did take the geographical aspects of depression into account had a cross-sectional design (Grigoroglou et al., 2020; Rivera & Mollalo, 2022), assessed depression by means of self-report and not an official medical diagnosis (Fernández-Niño et al., 2019), assessed a proxy of antidepressant prescription (Comber, Brunsdon, Charlton, & Cromby, 2021), relied on non-country-level data (Rodero-Cosano et al., 2016), or utilised conventional non-spatial regression and algorithmic models rather than spatial statistical methodologies that explicitly account for the spatial characteristics and distribution of data (Comber et al., 2021; Fernández-Niño et al., 2019; Grigoroglou et al., 2020). To our knowledge, the limited number of studies in the topic to date have not explored the spatio-temporal epidemiology of depression in a national data collection of medical records using spatio-temporal analysis methods. Hence, our current understanding of spatial patterns and trends of depression is limited.

Our understanding of depression influences how we prevent and treat the condition; therefore, improving our knowledge of the spatio-temporal dynamics of depression is paramount for identifying places in most need of help, based on their neighbourhood socioeconomic features (Fernández-Niño et al., 2019; Grigoroglou et al., 2020). Knowledge of the patterns and trends of depression can aid in the rapid evaluation of public health action in identified priority areas and the design of effective policies for tackling mental health inequalities (Grigoroglou et al., 2020).

To address this gap in knowledge, we conducted a longitudinal spatial study of depression with two main objectives: firstly, to examine the spatio-temporal patterns and trends of diagnosed depression among adults in England in a longitudinal spatial study design, and secondly, to evaluate the influence of neighbourhood socioeconomic deprivation in explaining these patterns.

## 2 Materials and methods

### 2.1 Data sources

The unit of analysis in all geospatial models was the Lower Super Output Area (LSOA). There are 32,844 LSOAs across England, with an average population of 1500 people (Office for National Statistics, 2021). In all analyses, we used the LSOA boundaries published by the Office for National Statistics as at March 21, 2021 (Office for National Statistics, 2021). In addition, we used the digital vector boundaries for Government Office Regions (GOR) to allow for comparisons within the highest tier of sub-national division in England (North East, North West, Yorkshire and the Humber, East Midlands, West Midlands, East of England, London, South East, and South West).

The diagnosed depression prevalence was derived using the data published by NHS Digital. Figures showing the recorded prevalence of depression in England by general practitioner (GP) practice are published annually in the Quality and Outcomes Framework (QOF) administrative dataset, which also reports how the QOF-recorded prevalence has changed since the previous year (NHS Digital, 2020, pp. 2019–2020). For this study, we combined all available data on depression published by NHS Digital and created time-series recorded depression for each LSOA from 2011 to 2022. The annual aggregate data on diagnoses of depression per LSOA has been calculated based on the weighted averages of the number of patients diagnosed with depression per LSOA divided by the total number of registered patients in each LSOA. In terms of coverage, the data for Quality and Outcomes Framework (QOF) have been collected annually at an aggregate level for each of the 6470 (97.5%) GP practices in England, with approximately 61 million registered patients aged 18 years and above; thus, the dataset offers nationwide insights.

The Index of Multiple Deprivation (IMD) is a widely used statistic within the UK to classify the relative deprivation of small areas census geographies (Abel, Barclay, & Payne, 2016). In our analyses, we used the latest English Index of Multiple Deprivation (IMD 2019), in which the following seven domains of deprivation are considered and weighted with different strengths and compiled into a single score of deprivation: income, employment, education, health, crime, barriers to housing and services, and living environment (McLennan et al., 2019).

### 2.2 Analytical approach

In a univariate analysis, the recorded prevalence of depression was described each year with minimum and maximum values, central tendency measures (mean and median), and dispersion measures (range, standard deviation and variance). To measure spatial autocorrelation across the region, we applied the Global Moran’s I statistic (Getis & Ord, 1992). The conceptualisation of Spatial Relationship was set as ‘Contiguity edges corners’ and the standardisation parameter was set us ‘Row’. This measured spatial autocorrelation for each year using LSOA and values of depression simultaneously and evaluated whether the pattern expressed was clustered, dispersed, or random (Getis & Ord, 1992). The Global Moran’s I null hypothesis states that the attributes being analysed are spatially uncorrelated.

Guided by the results of Global Moran’s I, we applied Cluster and Outlier Analysis, using the Anselin Local Moran’s I algorithm (Anselin, 1995), to identify local indicators of spatial association (LISA) and correct for spatial dependence. The conceptualisation of spatial relationships parameter value was set as the ‘Contiguity edges corners’, the standardisation option was set as ‘Row’, and the number of permutations was set as 999. The LISA refer to statistically significant spatial clusters of small areas with high values (high/high clusters) and low values (low/low clusters) of depression, as well as high and low spatial outliers in which a high value is surrounded by low values (high/low clusters), and outliers in which a low value is surrounded by high values (low/high clusters), corrected for multiple testing and spatial dependence using the False Discovery Rate (FDR) correction method.

We extended our spatial methods, including space-time pattern mining analysis (Naqvi et al., 2021) in the recorded prevalence of depression per LSOA, for which there were no missing values. We performed by creating a data structure that summarises the data into three-dimensional space-time bins, that show the absolute location (x and y dimensions) and absolute time (z dimension) simultaneously. Finally, the points were counted for each bin and summary field value trends were evaluated for all bin locations. Summarised data were used as input for the space-time pattern mining analytics; in that analysis, we performed a longitudinal space-time implementation of the Anselin Local Moran’s I statistic to examine statistically significant clusters and outliers in the context of both space and time (Naqvi et al., 2021).

Next, we created a collection of clusters based on the similarity of time series values. The time series were clustered based on their similarity of value, with approximately equal values across time, indicating a high, medium and low rate of increase.

To assess spatial nonstationarity between IMD and depression, we used the Koenker (BP) Statistic. This test determines whether the relationship under examination changes across the geographic space (Naqvi et al., 2021). To determine the overall model significance, we consulted the Joint Wald Statistic.

Due to statistically significant spatial nonstationarity, Geographically Weighted Regression (GWR) (Naqvi et al., 2021) was applied as the most appropriate method to understand the local effects of the latest available data on relative deprivation (IMD 2019) on recorded depression in 2020, with a focus on achieving temporal alignment (Comber et al., 2021). GWR is a local regression model that constructs a single equation for each feature in the study area using only its neighbouring features and allows for variable relationships to change over space. Therefore, we calculated the local R-squared for each feature in the study area.

Statistical significance was set at the 99% confidence level to enhance the robustness and stringency of our results. Analyses were performed in ArcGIS Pro Version 2.9.2 (Esri Inc., 2021) using the following tools, in order of execution: Spatial Join tool, Spatial Autocorrelation (Global Moran's I) tool, Optimised Outlier Analysis tool (999 permutations), Space-Time Cube Creation tool, Space-Time Pattern Analysis tool, Time Series Clustering tool and Tabulate Intersection tool.

## 3 Results

Table 1 shows the summary statistics of the recorded prevalence of depression in England from 2011 to 2022. The absolute range of depression prevalence among LSOAs in England increased from 15.42 to 26.67 from 2011 to 2022. Also, the variance increased from 3.76 to 10.92 over the study period, indicating a rise in the spread of prevalence scores from the mean value in each consequent year.

**Table 1:** Summary statistics of recorded depression prevalence in England from 2011 to 2022.^a^

Year	Min	Max	Mean	Median	Range	Standard Deviation	Variance
2011	0.31	15.73	5.84	5.79	15.42	1.94	3.76
2012	0.36	15.98	6.09	6.04	15.62	1.92	3.68
2013	0.64	17.87	5.90	5.75	17.23	1.80	3.25
2014	0.99	16.34	6.60	6.46	15.35	1.98	3.91
2015	1.04	17.44	7.43	7.30	16.41	2.18	4.74
2016	1.38	21.66	8.37	8.21	20.28	2.33	5.41
2017	1.87	22.85	9.25	9.05	20.98	2.50	6.26
2018	2.05	24.20	10.05	9.85	22.16	2.70	7.32
2019	1.72	39.55	10.98	10.75	37.83	2.94	8.63
2020	2.22	28.75	11.80	11.57	26.53	3.14	9.84
2021	2.54	28.70	12.53	12.29	26.16	3.27	10.68
2022	3.09	29.76	12.94	12.72	26.67	3.30	10.92

a The dataset on Quality and Outcomes Framework Indicators: Depression prevalence (QOF_4_12) Version 1.00 (Daras et al., 2023).

The results of the Global Moran’s I statistic that assess the overall pattern and trend of the data from 2011 to 2022 are shown in Table 2. The Global Moran’s I statistic for 2011–2012 was stable at around 0.86, whereas it slightly decreased in 2013 and gradually increased by 0.01 points every year to reach 0.91 in 2022. The p-value for the Global Moran's I value was statistically significant, and the z-score was positive for all years from 2011 to 2022.

**Table 2:** Global Moran’s I statistic of recorded depression prevalence in England from 2011 to 2022.^a^

Year	Index	Z-score	P-value
2011	0.864	259.269	0.000000
2012	0.867	260.229	0.000000
2013	0.839	251.907	0.000000
2014	0.849	254.680	0.000000
2015	0.856	256.842	0.000000
2016	0.865	259.680	0.000000
2017	0.877	263.269	0.000000
2018	0.883	264.575	0.000000
2019	0.892	267.651	0.000000
2020	0.900	270.233	0.000000
2021	0.905	271.651	0.000000
2022	0.906	271.945	0.000000

a The dataset on Quality and Outcomes Framework Indicators: Depression prevalence (QOF_4_12) Version 1.00 (Daras et al., 2023).

**Fig. 1:**
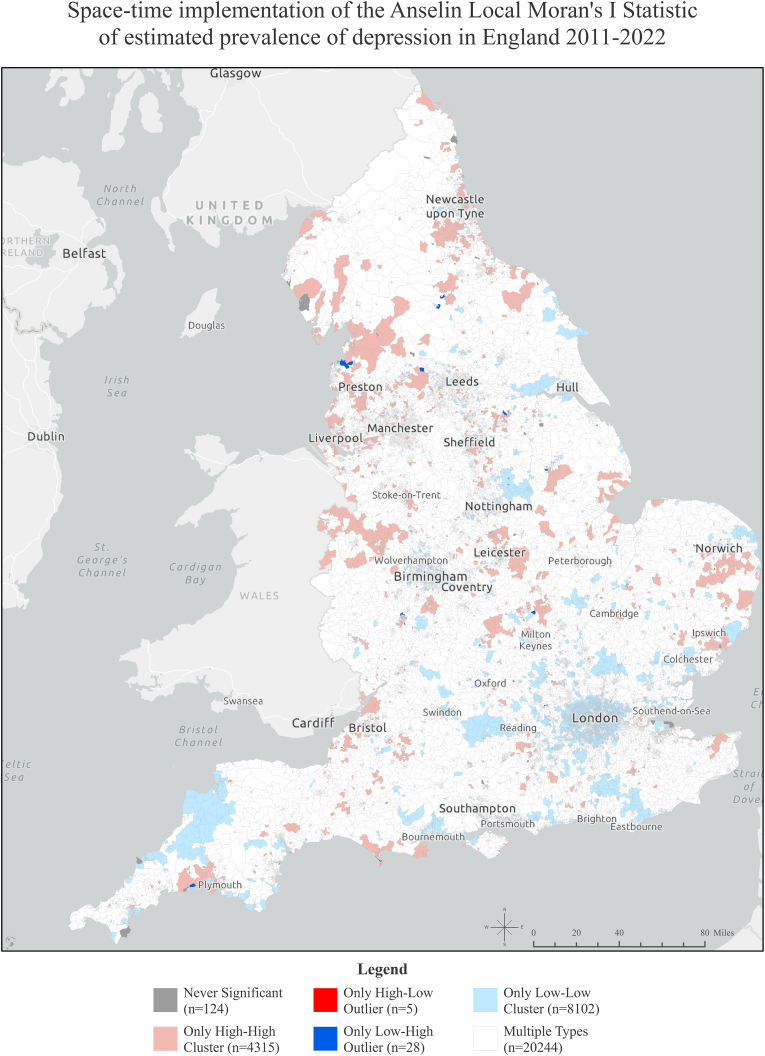
Space-Time Pattern Mining for recorded depression prevalence from 2011 to 2022, based on the Anselin Local Moran’s I algorithm.

The results of the Anselin Local Moran’s I algorithm of recorded depression prevalence from 2011 to 2022 are shown in Fig. S1 in the supplemental material. The LISA revealed statistically significant spatial clusters of depression with high values (high/high clusters) and low values (low/low clusters), as well as outliers (high/low and low/high). The longitudinal space-time implementation of the Anselin Local Moran’s I statistic is depicted in Fig. 1. The area coverage in m^2^, the estimated population residing, and the percentages of coverage and population per cluster in each region are shown in Table S1 in the supplemental material. The North West Region of England had the highest concentration of High-High clusters of depression, with 17.4% of the area having high values surrounded by high values in both space and time, and an estimated 24% of the population living in those areas (1.8 million people). It is worth mentioning that North East had a lower percentage of area coverage (10.2%), but a higher portion of the region’s population residing in high-high clusters (31%, n = 822,190 people). On the other hand, London was the region with the lowest percentage of the area (0.38%) and population (0.005%, n = 41,059) living in high-high clusters of recorded depression prevalence.

**Fig. 2:**
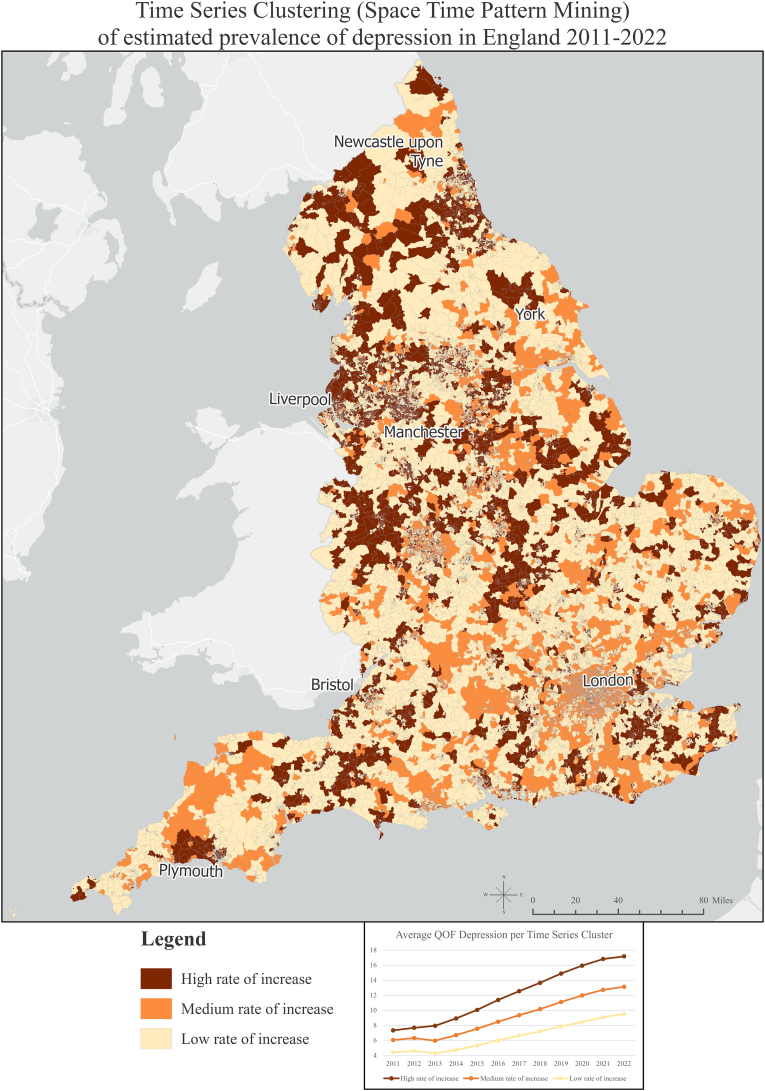
Map of England by Lower Super Output Areas (LSOAs), showing the results of the Time Series Clustering of the recorded depression prevalence from 2011 to 2022.

Fig. 2 shows the results of the Time Series Clustering tool, which created collections of clusters depicting the similarity in the trends of time-series values of the recorded prevalence of depression derived from the Anselin Local Moran’s I algorithm. We observed three distinct clusters of areas with statistically similar values over time, representing trends of high, medium, and low increase. The detailed characteristics of all clusters are presented in Tables S2 and S3 in the supplemental material. All areas experienced a prevalence of more than doubling from 2011 to 2022, as indicated by Prevalence Rate Ratios (PRR) (Zocchetti, Consonni, & Bertazzi, 1997) of 2.34 (99% CI 2.25–2.43), 2.17 (99% CI 2.10–2.23), and 2.15 (99% CI 2.07–2.23) for those with a high, medium or low rate of increase, respectively.

Inequalities widened among areas with a high rate of increase in recorded depression prevalence compared to those with a lower rate of increase, with the PRR increasing from 1.66 (99% CI 1.61–1.70) in 2011 to 1.81 (99% CI 1.76–1.85) by 2022. This demonstrates a significant broadening of mental health inequalities between areas with higher rates and those with lower rates over time. The area coverage in m^2^, the estimated population residing, and the percentages of coverage and population per cluster in each region are shown in Table S4 in the supplemental material. As shown in Table S2, the North West region had the greatest percentage of areas with a high rate of increase (43.1%), which might indicate that mental health inequalities are widening more rapidly in that region. Nearly half of the population in the North West (48.8%, n = 3,510,943 people) were living in an area with a high rate of increase, compared to 2.47% of the population in London (n = 221,651 people).

**Fig. 3:**
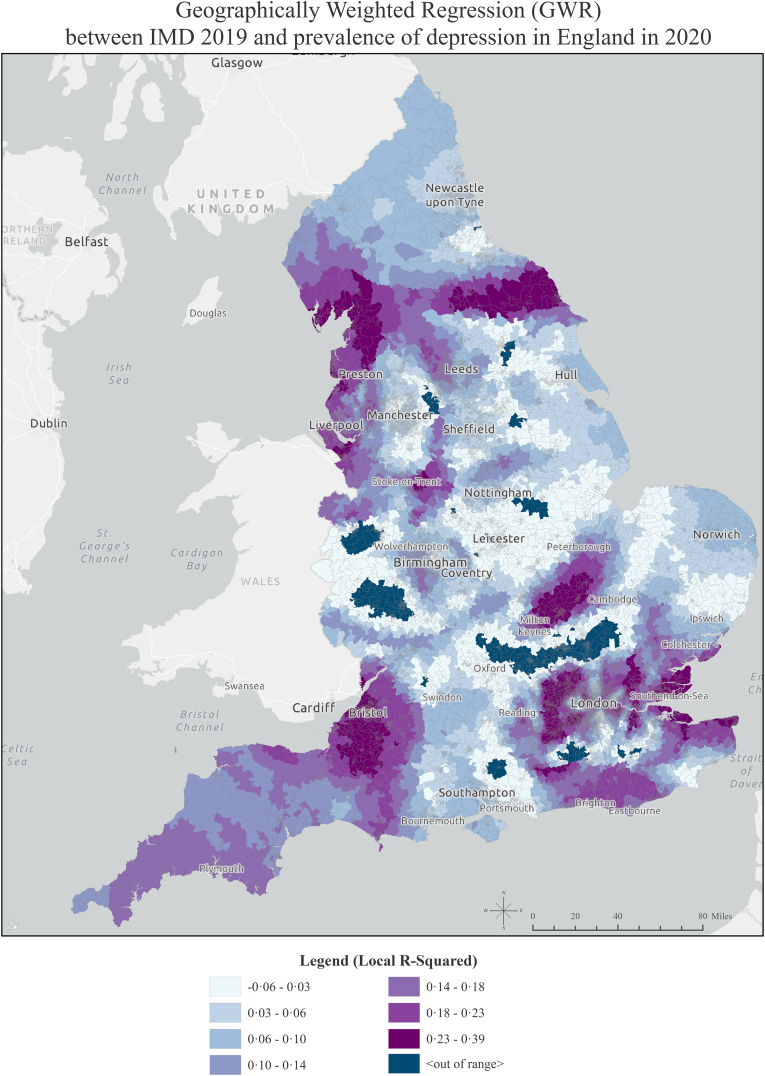
Map of England by Lower Super Output Areas (LSOAs), showing the results of Geographically Weighted Regression between Index of Multiple Deprivation (IMD) 2019 and recorded prevalence of depression in England in 2020.

The Koenker (BP) Statistic revealed statistically significant nonstationarity, indicating that the relationship between IMD and depression was different in different parts of the study area. The correlation coefficients scatter plot for all LSOAs is shown in Fig. S2 in the supplemental material. Fig. 3 illustrates the data on IMD19 and depression in 2020, using coefficients that indicate the proportion of the variance in recorded depression prevalence explained by IMD across different spatial locations. The darker areas do not indicate where there is the highest deprivation or highest depression prevalence; rather, they reveal where the relationship between IMD and depression is the strongest, informed by the results of GWR (Naqvi et al., 2021). As we see, the local R-squared varies across the country, which shows that IMD may be a strong predictor of depression in one area, explaining 3%–39% of the variance in depression in 2020 across the country. Summarised results of GWR between IMD 2019 and the recorded prevalence of depression in 2020 in each region are shown in Table S5 in the Supplemental Material.

## 4 Discussion

### 4.1 Summary of main findings

This study investigated spatio-temporal patterns and trends of depression in a national data collection of medical records in England using spatial statistics methodology. To our knowledge, this is the first longitudinal spatial analysis of depression at that national level, that used advanced spatial statistics to uncover spatio-temporal patterns and trends in depression. Between 2011 and 2022, the prevalence of recorded depression increased disparately across England, leading to a widening of mental health inequalities among small geographic areas. In the 12-year nationwide longitudinal spatial analysis of recorded rates of depression, we found that the observed values of increasingly diagnosed depression were not randomly distributed in the LSOAs but significantly spatially correlated; a phenomenon that become more pronounced over time.

The analysis revealed a North–South divide in mental health over time. North West and North East had the highest percentage of population residing in high-high clusters of depression. In addition, North West had the highest percentage of the population living in areas with a high increase trend in recorded depression prevalence, which might indicate that mental health inequalities are widening more rapidly in that region. In contrast, London had the lowest percentage of the population living in areas with both high-high clusters and a high rate of increase. Furthermore, our findings highlight the influence of neighbourhood socioeconomic deprivation in 2019 in shaping the spatial distribution of depression in the following year. The results of GWR revealed that South West, London and North West were the regions where the relationship between neighbourhood socioeconomic deprivation and depression was the strongest.

### 4.2 Comparison with previous literature

This study showed a continued increase in the prevalence of depression from 2011 to 2022, in line with global trends (GBD 2019 Mental Disorders Collaborators, 2022). Prior research has presented numerous potential explanations for the escalating prevalence of depression linked to the expanding burden of chronic diseases, proposing that this phenomenon arises from an evolutionary mismatch between past human environments and present-day living conditions. Among the proposed factors, declining social capital and increased socioeconomic inequality stand out as potential mediators contributing to a depressiogenic social milieu (Hidaka, 2012).

Studies have previously suggested that the recorded prevalence of severe mental illness in primary care in England is higher in the most deprived areas (Kontopantelis et al., 2015). Prior research has also commended that the austerity measures implemented following the recession that started in April 2008 have had different lasting negative impacts on mental health across groups (Kendrick, Stuart, Newell, Geraghty, & Moore, 2015), which were more damaging for those living in disadvantaged areas, where the impacts of both common and serious mental distress were disproportionately experienced (Barr et al., 2016; Iacobucci, 2014).

The current nationwide study builds upon this prior work and reveals a high global autocorrelation (Getis & Ord, 1992) of depression, indicating a violation of the fundamental assumption of data independence within the recorded depression prevalence dataset. In situations characterised by such spatial dependencies, conventional non-spatial regression methods become unsuitable, possibly leading to 'serious errors in model interpretation' (Getis & Ord, 1992). This limitation arises because global statistics are most effective when the spatial pattern is consistent across the entire study area. To address this challenge, our study incorporated local statistics, such as the Anselin Local Moran’s I, which evaluate each feature within the context of neighbouring features. This approach now offers a more nuanced assessment by comparing the local situation to the global situation, analogous to computing a mean or average for a set of values. A mean serves as a global statistic, providing an accurate representation if all values are clustered around a central value. However, if the dataset exhibits spatial heterogeneity, relying solely on a mean can yield misleading interpretations when values vary widely across different regions.

Previous time trend analyses of GP recording of depression in England have shown that depression is increasing; however, these studies explored data before 2013 (Kendrick et al., 2015) or applied standard regression and algorithmic models (Comber et al., 2021; Grigoroglou et al., 2020; Kendrick et al., 2015). As reported by Comber and colleagues (Comber et al., 2021), they examined the proxy of antidepressants by applying random forest models, an algorithmic procedure that does not take into consideration the spatial correlation. As they have acknowledged in their work, no national picture of depression exists in England that has examined the spatial characteristics and distribution of data, and commented on the necessity of considering these issues and quantifying the local mental health impacts of deprivation (Comber et al., 2021).

In a previous study conducted in England, researchers examined the relationship between IMD and depression using traditional regression models. However, they acknowledged potential limitations arising from the geographic heterogeneity that could affect the generalisability of their findings (Qi et al., 2022). Additionally, the variability in follow-up times across previous studies may contribute to the inconsistent evidence regarding the causal relationship between neighbourhood socioeconomic conditions and depression observed over the past few decades (Richardson, Westley, Gariépy, Austin, & Nandi, 2015). Understanding this relationship is complex due to the dynamic nature of neighbourhood socioeconomic conditions across space and variations in how these conditions are conceptualised. Moreover, individual-level measurements may not fully account for the characteristics and mobility aspects of the population, including those escaping concentrated disadvantage in either their immediate or surrounding neighbourhoods. This mobility can influence the neighbourhood conditions individuals live in, further complicating the analyses (Chyn & Katz, 2021; Cummins, Curtis, Diez-Roux, & Macintyre, 2007; Graif et al., 2016; Leventhal & Brooks-Gunn, 2003; Richardson et al., 2015). In such scenarios, standard regression techniques may yield biased results.

Our research contributes by being the first to employ spatial statistics to explore the locally varying effects of neighbourhood socioeconomic deprivation on recorded depression prevalence, a dimension not addressed in previous studies (Comber et al., 2021; Fernández-Niño et al., 2019; Grigoroglou et al., 2020). Our study emphasises the importance of checking for spatial autocorrelation, as its presence challenges the assumption of independence of residuals, raising concerns about the validity of hypothesis testing through conventional statistics (Lin & Wen, 2022). By utilising spatial methods, our study enhances the ability to capture and interpret the nuanced geographical influences on depression, providing now a more robust analytical framework for investigating complex spatial relationships in mental health. Our study, therefore, contributes to an already substantial body of research during the past half-century (Chyn & Katz, 2021) by advancing current knowledge through the elucidation of spatial and temporal trends in depression and provides a novel contribution by quantifying the localised spatial impacts of neighbourhood deprivation, a facet that has not been previously explored in this context.

### 4.3 Strengths and limitations

A key strength of this study is that it was conducted with a large database containing aggregated data on diagnoses of depression for a national population of over 61 million people using the same classification system across areas. Another strength was that the latest tools of the ArcGIS Pro Version 2.9.2 (Esri Inc., 2021) were used, which allowed advanced space-time patterns mining analysis and geoprocessing (Lin & Wen, 2022; Naqvi et al., 2021). Using spatial methodology allowed the identification of spatial clustering patterns, detection of localised hot spots and identification of local risk factors, which is not feasible through traditional non-spatial regression models. In our analyses, we employed the LSOA boundaries published by the Office for National Statistics as of 21st March 2021 (Office for National Statistics, 2021), which are the same as the Quality and Outcomes Framework Indicators boundaries. Nevertheless, we acknowledge that the fixed nature of these boundaries may not fully capture the dynamic aspect of the relationship between health and place, a limitation inherent in all studies that rely on fixed boundaries.

It is important to acknowledge the limitations of our findings before considering their implications. The study explored the overall trends of depression that drive the local public health costs without accounting for underlying causes each year. However, we should note that within local areas, the portion of the most vulnerable population (e.g., unemployed or with pre-existing mental health problems) may vary from time to time (Edwards, 2008).

Moreover, the risk associated with residing in a community within the highest quintile of community-level deprivation may differ based on race and ethnicity (Lo et al., 2021). Unfortunately, it was not feasible to incorporate this aspect into our analyses to assess its impact on recorded depression prevalence based in the data provided by NHS Digital (Daras, Rose, Tsimpida, & Barr, 2023).

A limitation of the dataset stems from a change in the definition of depression prevalence in the QOF database from 2012 to 2013. Before 2013, the definition was ‘Patients with a history of depression coded at any time’, while from 2013 onwards, it became ‘Patients with a history of depression since April 2006.’ Although the discontinuity in depression prevalence between the years 2011–2012 and post-2013 has been addressed through the statistical adjustment of slopes for the respective periods (Daras et al., 2023), this alteration in the definition may account for the slight decrease observed in the Global Moran's I statistic for 2013.

Moreover, there are limitations to the diagnosis of depression in primary care, including the possibility that GPs may not identify all patients who could potentially benefit from treatment, leading to incomplete or inconsistent clinical data records (Kontopantelis et al., 2015). The GPs’ decisions to diagnose depression may also be influenced by personal bias or preferences, as they may record symptoms rather than a formal diagnosis of depression (Grigoroglou et al., 2020).

Additional significant factors include stigma and the cultural interpretation of symptom clusters as indicative of depression rather than alternative explanations. This may influence individuals' willingness to present mental health problems to healthcare providers, accept a diagnosis, and may introduce variations in demographic factors, such as differences among various ethnicities, impacting the diagnoses and recording methods (Kontopantelis et al., 2015). Moreover, socioeconomic and cultural disparities may contribute to varying attitudes towards mental health, influencing the availability of resources, constraints in seeking medical treatment, and the dynamics of self-diagnosis and formal diagnosis by a health professional.

The potential for self-referral to NHS therapy services should feed through with the diagnosis of depression to the GP record. Therefore, overall, the recorded prevalence of depression recorded in the QOF is likely an underestimation of the actual prevalence of depression in the population (Grigoroglou et al., 2020). Another limitation is that the QOF depression prevalence presents aggregate data for all adults without any information on adolescents or specific age groups or information on those in retirement status, which may confound the associations (Tsimpida, Kontopantelis, Ashcroft, & Panagioti, 2022).

We acknowledge another limitation in our study, specifically regarding the analysis of the relationship between IMD and depression. While we achieved temporal alignment, it is important to note that our analysis was not longitudinal. This lack of a longitudinal approach restricts the generalisability of findings about the relationship between depression and IMD across different spatial contexts. In light of this limitation, we advocate for future research, particularly when an updated Index of Multiple Deprivation (IMD) becomes available. The forthcoming release of an updated IMD, with a provisional date set for late 2025 (Department for Levelling Up, Housing and Communities, 2022) underscores the need for continued investigation to further enhance our understanding of the dynamics between depression and deprivation over time and space.

### 4.4 Research and policy implications

This study holds significant implications for future research. Our findings highlight that the prevalence of depression is influenced by the spatial distribution of high and/or low values within the dataset, suggesting a nuanced relationship between depression rates and neighbourhood characteristics. Understanding neighbourhood and place effects has been a prominent inquiry for social scientists over the past fifty years. Recent empirical studies employing experimental and quasi-experimental research designs have provided fresh insights into the significance of residential neighbourhoods in shaping mental health outcomes. Our research provides new insights into experimental and quasi-experimental research designs, particularly in the context of mental health research. In instances where evidence is generated through randomised control trials (RCTs) that may not have accounted for the role of location, it becomes imperative to carefully consider contextual factors beyond individual characteristics by employing spatial modelling techniques. Despite being considered gold standards (Grossman & Mackenzie, 2005), RCTs may not be adequate in capturing changes in mental health outcomes pre-and post-interventions unless nuanced spatial influences are taken into account. Presently, RCTs do not consider contextual factors related to geographic location; instead, they focus solely on the individual characteristics of the two (or more) groups in a trial that should be as similar as possible. This narrow focus may potentially lead to unintentionally misleading interpretations. For instance, the effects of the same treatment on the mental health of participants with similar characteristics can be either amplified or masked based on differences in the ‘spatial mental burden’ of the places they reside.

The growing spatial autocorrelation we observed in recorded depression prevalence might be attributed to social and environmental influences, as well as residential segregation. This phenomenon may occur because populations with similar characteristics residing in close proximity are likely to share common experiences and challenges related to mental health. The role of social fragmentation necessitates further investigation, as also highlighted by (Grigoroglou et al., 2020). We strongly advocate for more research in the field of socio-spatial mental health inequalities to investigate potential mechanisms.

In our study, deprivation explained up to 0.39 of the variance in the recorded depression prevalence in 2020, which varied spatially across the country. More research is needed to understand localised social, economic and environmental mechanisms, which were beyond this study’s scope. Further research should also compare small areas where deprivation plays a higher role in explaining high recorded depression prevalence against those where deprivation does not explain much of the variance in depression and areas with high neighbourhood deprivation but low levels of recorded depression prevalence. Such an analysis may illuminate how localised factors distinctly shape the strength of the relationship between IMD and depression. Furthermore, recognising that IMD may moderate the effect of other risk factors, a more nuanced understanding of these interactions is crucial for advancing our comprehension of local mental health dynamics.

This level of understanding can shape evidence-informed public mental health interventions beyond the prescription of antidepressant drugs. Identifying and monitoring the high-priority small areas will allow for better allocation of resources. However, more research is needed to investigate the relationship between depression and risk factors that may exacerbate mental health outcomes in specific geographic areas. Potential areas for further investigation are neighbourhood attributes measuring the role of green space (Lee & Maheswaran, 2011), and other characteristics of the living environment such as crime statistics, educational prospects (Baranyi, Di Marco, Russ, Dibben, & Pearce, 2021), levels of safety, physical hazards, pollution and levels of social support (Park et al., 2021).

Furthermore, depression is an umbrella condition which contains subtypes; future research could consider specific types of depression, for example, prenatal and postnatal depression; further research will help understand the variation in rates across different areas and how it relates to other health conditions that may exacerbate mental health inequalities, such as hearing loss (Tsimpida, Kontopantelis, et al., 2022; Tsimpida, Panagioti, & Kontopantelis, 2022). The study of how neighbourhoods may affect the mental health of older adults and young people is a growing area of research (Corcoran et al., 2022; Park et al., 2021), which suggests that treating depression may involve addressing not only individual issues (Marwaha et al., 2022) but also the characteristics of the neighbourhood in which a person lives. Furthermore, long-term exposure to a neighbourhood per se may impact people’s mental health outcomes (Park et al., 2021). This is a useful insight for further study from a life-course epidemiological perspective and might explain why previous studies exploring mental health outcomes in different areas have produced inconsistent findings. It may be the case that the mixed evidence in the literature is due to spatial heterogeneity of mental health data according to the length of follow-up in a specific area (Richardson et al., 2015), as people’s neighbourhood conditions can change over time.

The findings may have important policy implications: the study of the spatial structure of mental disorders is particularly important to inform public health interventions at a national or provincial level. Such approaches could lead to developing strategic health and non-health programs; knowing where relationships between neighbourhood socioeconomic deprivation and depression are the strongest can help in focusing on any remediation efforts or deciding how to address the problem.

The findings may also have implications for practice. The National Institute for Clinical Excellence (NICE) guidelines recommend a comprehensive biopsychosocial approach to tackle the burden of depression (National Institute for Clinical Excellence, 2022). Since we found that in some areas of England, deprivation plays a more prominent role in depression rates, a novel socio-spatial approach is needed to understand the population’s mental health. These findings suggest that place-related characteristics, not individual propensity towards developing mental health issues, may explain the mental health burden in the country and the exaggeration of mental inequalities. Therefore, tackling mental health inequalities may rest on making social, economic and environmental changes to prevent or treat depression.

## 5 Conclusions

This study highlighted the increasing mental health inequalities in England from 2011 to 2022 and emphasised the need to investigate geographic inequalities using spatial modelling. By combining spatial statistics and GIS, our study first showed new opportunities for identifying areas with persistently high or increasing levels of recorded depression prevalence and identifying the impact of deprivation on depression rates. A socio-spatial approach can help identify and monitor high-priority areas and allocate resources effectively, providing policymakers and public health professionals with vital insights to reduce the burden of mental health disorders. The knowledge gained from this analysis can benefit public health professionals and policymakers worldwide. Targeted prevention and intervention strategies have the potential to facilitate prompt evaluation of public health measures in priority areas and provide guidance for medical and non-medical public health interventions that are sensitive to local contexts and take into account the ‘spatial mental burden’ and local parameters of places where people reside.

## Financial disclosure statement

This research received no specific grant from any funding agency in the public, commercial, or not-for-profit sectors. KD, RC, and MG are part funded by the National Institute for Health and Care Research Applied Research Collaboration North West Coast (ARC NWC). The views expressed herein are those of the authors, not necessarily the Institute for Health and Care Research nor the Department for Health and Social Care.

## Ethical statement

The dataset on Quality and Outcomes Framework Indicators: Depression prevalence (QOF_4_12) is publicly available in the Place-based longitudinal data resource (Original record link: https://pldr.org/dataset/2ldz5, Data catalogue DOI: 10.17638/datacat.liverpool.ac.uk/2170). According to the corresponding author's institution, a secondary analysis of publicly available data does not require Institutional Review Board approval.

## CRediT authorship contribution statement

**Dialechti Tsimpida:** Writing – review & editing, Writing – original draft, Visualization, Validation, Supervision, Software, Resources, Project administration, Methodology, Investigation, Formal analysis, Data curation, Conceptualization. **Anastasia Tsakiridi:** Writing – review & editing, Writing – original draft, Visualization, Validation, Methodology, Investigation, Formal analysis, Data curation, Conceptualization. **Konstantinos Daras:** Writing – review & editing, Methodology, Investigation, Data curation. **Rhiannon Corcoran:** Writing – review & editing, Investigation. **Mark Gabbay:** Writing – review & editing, Investigation.

## Declaration of competing interest

None.

## Data Availability

The dataset on Quality and Outcomes Framework Indicators: Depression prevalence (QOF_4_12) Version 1.00 is available at Quality and Outcomes Framework Indicators: Depression prevalence (QOF_4_12) (Reference data) (DataCat: The Research Data Catalogue) (Daras et al., 2023).
