# Supplementary material for: Unravelling the dynamics of mental health inequalities in England: A 12-year nationwide longitudinal spatial analysis of recorded depression prevalence
Source: SSM Popul Health. 2024 Apr 15;26:101669. doi: 10.1016/j.ssmph.2024.101669 (PMC11066558; doi:10.1016/j.ssmph.2024.101669)
Supplement: Supplementary file [file mmc1.docx]

**Supplemental Material**

Table of contents

**Table S1. Area coverage and estimated population residing in Regions of England based on the space-time pattern mining (Anselin Local Moran's I Statistic) of the recorded prevalence of depression in Lower Super Output Areas (LSOA) from 2011 to 2022**……………………………..………………………… (page 2)

**Table S2. Characteristics of clusters of high, medium and low rate of increase and prevalence rate ratio in the recorded depression prevalence in England from 2011-2022.**

**Table S3. Summary statistics of clusters of high, medium and low rate of increase in the recorded depression prevalence in England from 2011-2022.**

**Table S4. Area coverage and estimated population residing in Regions of England based on the Time Series Clustering of the recorded prevalence of depression in Lower Super Output Areas (LSOA) from 2011 to 2022**. ………..……………………………..……………………………………………………… (page 4)

**Table S5. Results of Geographically Weighted Regression between Index of Multiple Deprivation (IMD) 2019 and recorded prevalence of depression in England in 2020**………………………………………..……… (page 5)

**Figure S1. Maps of England by Lower Super Output Areas (LSOAs), showing the results of Anselin Local Moran’s I algorithm for recorded depression prevalence from 2011-2022**. …………………………………… (page 6)

**Figure S2. Correlation coefficients scatter plot between Index of Multiple Deprivation (IMD) scores* in 2019 and Depression in 2020 in all Lower Super Output Areas (LSOAs) in England (y = 10.8 + 0.046 x)**

*The exponential transformation for the production of IMD scores is described in detail in the following link:<https://assets.publishing.service.gov.uk/government/uploads/system/uploads/attachment_data/file/833951/IoD2019_Technical_Report.pdf> ……………………………………………….…………….……..……… (page 7)

**Table S1. Area coverage and estimated population residing in Regions of England based on the space-time pattern mining (Anselin Local Moran's I Statistic) of the estimated prevalence of depression in Lower Super Output Areas (LSOA) from 2011 to 2022.**

| **Num** | **Region** | **Category** | **Area km^2^** | **Percentage of area** | **Estimated population residing in these areas*** | **Percentage of the population in the region** |
| --- | --- | --- | --- | --- | --- | --- |
| 1 | East Midlands | Multiple Types | 13,167.94 | 84.12 | 3,240,370 | 0.67 |
|  |  | Never Significant | 20.12 | 0.13 | 17,775 | 0.00 |
|  |  | Only High-High Cluster | 1,745.81 | 11.15 | 907,170 | 0.19 |
|  |  | Only High-Low Outlier | 0.33 | 0.00 | 1,480 | 0.00 |
|  |  | Only Low-High Outlier | 10.51 | 0.07 | 8,591 | 0.00 |
|  |  | Only Low-Low Cluster | 693.22 | 4.43 | 684,333 | 0.14 |
| 2 | East of England | Multiple Types | 16,013.79 | 83.59 | 4,275,687 | 0.68 |
|  |  | Never Significant | 25.64 | 0.13 | 46,345 | 0.01 |
|  |  | Only High-High Cluster | 1,263.81 | 6.60 | 509,043 | 0.08 |
|  |  | Only High-Low Outlier | 0.32 | 0.00 | 1,682 | 0.00 |
|  |  | Only Low-Low Cluster | 1,775.71 | 9.27 | 1,412,183 | 0.23 |
| 3 | London | Multiple Types | 411.89 | 26.04 | 1,812,794 | 0.20 |
|  |  | Never Significant | 0.39 | 0.02 | 5,035 | 0.00 |
|  |  | Only High-High Cluster | 5.97 | 0.38 | 41,059 | 0.00 |
|  |  | Only High-Low Outlier | 0.00 | 0.00 | 0 | 0.00 |
|  |  | Only Low-Low Cluster | 1,153.08 | 72.91 | 7,111,012 | 0.79 |
| 4 | North East | Multiple Types | 7,573.91 | 87.99 | 1,662,715 | 0.62 |
|  |  | Never Significant | 40.56 | 0.47 | 18,978 | 0.01 |
|  |  | Only High-High Cluster | 877.10 | 10.19 | 822,190 | 0.31 |
|  |  | Only Low-High Outlier | 0.55 | 0.01 | 3,062 | 0.00 |
|  |  | Only Low-Low Cluster | 80.39 | 0.93 | 154,554 | 0.06 |
| 5 | North West | Multiple Types | 11,424.18 | 80.56 | 5,288,840 | 0.72 |
|  |  | Never Significant | 95.63 | 0.67 | 16,979 | 0.00 |
|  |  | Only High-High Cluster | 2,463.74 | 17.37 | 1,785,056 | 0.24 |
|  |  | Only High-Low Outlier | 0.08 | 0.00 | 989 | 0.00 |
|  |  | Only Low-High Outlier | 35.23 | 0.25 | 5,356 | 0.00 |
|  |  | Only Low-Low Cluster | 85.19 | 0.60 | 205,101 | 0.03 |
| 6 | South East | Multiple Types | 15,861.67 | 83.01 | 6,579,188 | 0.72 |
|  |  | Never Significant | 43.89 | 0.23 | 30,684 | 0.00 |
|  |  | Only High-High Cluster | 559.00 | 2.93 | 652,417 | 0.07 |
|  |  | Only Low-High Outlier | 2.34 | 0.01 | 4,959 | 0.00 |
|  |  | Only Low-Low Cluster | 2,577.59 | 13.49 | 1,896,991 | 0.21 |
| 7 | South West | Multiple Types | 19,388.46 | 80.84 | 3,835,537 | 0.68 |
|  |  | Never Significant | 87.20 | 0.36 | 33,603 | 0.01 |
|  |  | Only High-High Cluster | 1,400.92 | 5.84 | 820,522 | 0.15 |
|  |  | Only High-Low Outlier | 0.53 | 0.00 | 1,512 | 0.00 |
|  |  | Only Low-High Outlier | 11.69 | 0.05 | 0 | 0.00 |
|  |  | Only Low-Low Cluster | 2,893.63 | 12.06 | 929,540 | 0.17 |
| 8 | West Midlands | Multiple Types | 11,105.84 | 85.37 | 4,133,949 | 0.69 |
|  |  | Never Significant | 3.02 | 0.02 | 3,222 | 0.00 |
|  |  | Only High-High Cluster | 1,427.51 | 10.97 | 747,461 | 0.13 |
|  |  | Only Low-High Outlier | 5.45 | 0.04 | 6,018 | 0.00 |
|  |  | Only Low-Low Cluster | 451.01 | 3.47 | 1,082,434 | 0.18 |
| 9 | Yorkshire and The Humber | Multiple Types | 12,903.87 | 83.63 | 4,002,798 | 0.72 |
|  |  | Never Significant | 15.58 | 0.10 | 15,806 | 0.00 |
|  |  | Only High-High Cluster | 1,523.80 | 9.88 | 751,233 | 0.14 |
|  |  | Only High-Low Outlier | 0.27 | 0.00 | 1,758 | 0.00 |
|  |  | Only Low-High Outlier | 29.85 | 0.19 | 17,259 | 0.00 |
|  |  | Only Low-Low Cluster | 920.59 | 5.97 | 732,964 | 0.13 |

***** Based on Population Estimates for Lower Layer Super Output Areas in England <https://www.ons.gov.uk/peoplepopulationandcommunity/populationandmigration/populationestimates/datasets/lowersuperoutputareamidyearpopulationestimates>

**Table S2. Characteristics of clusters of high, medium and low rate of increase and prevalence rate ratio in the recorded depression prevalence in England from 2011-2022.**

|  | 2011 | 2012 | 2013 | 2014 | 2015 | 2016 | 2017 | 2018 | 2019 | 2020 | 2021 | 2022 |
| --- | --- | --- | --- | --- | --- | --- | --- | --- | --- | --- | --- | --- |
| High rate of increase | 7.33 | 7.67 | 7.94 | 8.91 | 10.05 | 11.36 | 12.54 | 13.64 | 14.88 | 15.93 | 16.81 | 17.16 |
| Medium rate of increase | 6.04 | 6.30 | 5.96 | 6.70 | 7.54 | 8.47 | 9.34 | 10.15 | 11.10 | 11.97 | 12.71 | 13.11 |
| Low rate of increase | 4.42 | 4.58 | 4.28 | 4.74 | 5.31 | 5.98 | 6.63 | 7.19 | 7.86 | 8.44 | 9.07 | 9.50 |
| Prevalence rate ratio | 1.66 | 1.67 | 1.86 | 1.88 | 1.89 | 1.90 | 1.89 | 1.90 | 1.89 | 1.89 | 1.85 | 1.81 |

**Table S3. Summary statistics of clusters of high, medium and low rate of increase in the recorded depression prevalence in England from 2011-2022.**

|  | Average value of the cluster | Median value of the cluster | Min value of the cluster | Max value of the cluster | Range value of the cluster | Standard Deviation value of the cluster |
| --- | --- | --- | --- | --- | --- | --- |
| High rate of increase | 12.02 | 11.95 | 7.33 | 17.16 | 9.84 | 3.66 |
| Medium rate of increase | 9.12 | 8.91 | 5.96 | 13.11 | 7.16 | 2.67 |
| Low rate of increase | 6.50 | 6.30 | 4.28 | 9.50 | 5.22 | 1.89 |

**Table S4. Area coverage and estimated population residing in Regions of England based on the Time Series Clustering of the estimated prevalence of depression in Lower Super Output Areas (LSOA) from 2011 to 2022.**

| **Num** | **Region** | **Category** | **Area km^2^** | **Percentage** | **Estimated population residing in these areas*** | **Percentage of the population in the region** |
| --- | --- | --- | --- | --- | --- | --- |
| 1 | East Midlands | Low rate of increase | 6,924.72 | 44.24 | 2,186,817 | 45.00 |
|  |  | Medium rate of increase | 2,904.74 | 18.56 | 1,133,973 | 23.33 |
|  |  | High rate of increase | 5,808.48 | 37.11 | 1,538,929 | 31.67 |
| 2 | East of England | Low rate of increase | 11,809.60 | 61.65 | 3,460,966 | 55.42 |
|  |  | Medium rate of increase | 4,784.74 | 24.98 | 1,836,113 | 29.40 |
|  |  | High rate of increase | 2,484.93 | 12.97 | 947,859 | 15.18 |
| 3 | London | Low rate of increase | 274.06 | 17.33 | 1,569,838 | 17.50 |
|  |  | Medium rate of increase | 1,273.58 | 80.53 | 7,178,411 | 80.03 |
|  |  | High rate of increase | 23.69 | 1.50 | 221,651 | 2.47 |
| 4 | North East | Low rate of increase | 4,672.14 | 54.28 | 1,187,939 | 44.63 |
|  |  | Medium rate of increase | 1,152.50 | 13.39 | 334,827 | 12.58 |
|  |  | High rate of increase | 2,747.87 | 31.92 | 1,138,733 | 42.79 |
| 5 | North West | Low rate of increase | 6,769.53 | 47.74 | 3,005,562 | 41.16 |
|  |  | Medium rate of increase | 1,227.13 | 8.65 | 785,816 | 10.76 |
|  |  | High rate of increase | 6,107.40 | 43.07 | 3,510,943 | 48.08 |
| 6 | South East | Low rate of increase | 9,860.98 | 51.60 | 4,776,583 | 52.12 |
|  |  | Medium rate of increase | 6,058.75 | 31.71 | 2,789,768 | 30.44 |
|  |  | High rate of increase | 3,124.77 | 16.35 | 1,597,888 | 17.44 |
| 7 | South West | Low rate of increase | 13,726.14 | 57.23 | 2,960,472 | 52.65 |
|  |  | Medium rate of increase | 5,805.53 | 24.21 | 1,282,832 | 22.82 |
|  |  | High rate of increase | 4,250.76 | 17.72 | 1,379,105 | 24.53 |
| 8 | West Midlands | Low rate of increase | 7,331.34 | 56.35 | 2,876,587 | 48.16 |
|  |  | Medium rate of increase | 2,042.56 | 15.70 | 1,658,330 | 27.76 |
|  |  | High rate of increase | 3,618.94 | 27.82 | 1,438,167 | 24.08 |
| 9 | Yorkshire and The Humber | Low rate of increase | 9,231.02 | 59.82 | 2,864,917 | 51.88 |
|  |  | Medium rate of increase | 3,161.41 | 20.49 | 1,311,795 | 23.76 |
|  |  | High rate of increase | 3,001.54 | 19.45 | 1,345,106 | 24.36 |

***** Based on Population Estimates for Lower Layer Super Output Areas in England <https://www.ons.gov.uk/peoplepopulationandcommunity/populationandmigration/populationestimates/datasets/lowersuperoutputareamidyearpopulationestimates>

**Table S5. Results of Geographically Weighted Regression between Index of Multiple Deprivation (IMD) 2019 and prevalence of depression in England in 2020.**

| **Num** | **Region** | **Mean Local R-squared** | **Median Local R-squared** | **Min Local R-squared** | **Max Local R-squared** | **Range Local R-squared** | **Standard Deviation Local R-squared** |
| --- | --- | --- | --- | --- | --- | --- | --- |
| 1 | East Midlands | 0.04 | 0.04 | -0.16 | 0.26 | 0.42 | 0.05 |
| 2 | East of England | 0.11 | 0.10 | -0.31 | 0.37 | 0.68 | 0.13 |
| 3 | London | 0.15 | 0.16 | -0.04 | 0.35 | 0.39 | 0.07 |
| 4 | North East | 0.05 | 0.05 | -0.07 | 0.11 | 0.18 | 0.03 |
| 5 | North West | 0.12 | 0.12 | -0.05 | 0.30 | 0.35 | 0.08 |
| 6 | South East | 0.11 | 0.10 | -0.27 | 0.39 | 0.66 | 0.10 |
| 7 | South West | 0.15 | 0.15 | -0.06 | 0.27 | 0.33 | 0.07 |
| 8 | West Midlands | 0.07 | 0.08 | -0.24 | 0.28 | 0.52 | 0.07 |
| 9 | Yorkshire and The Humber | 0.08 | 0.07 | -0.15 | 0.30 | 0.46 | 0.07 |


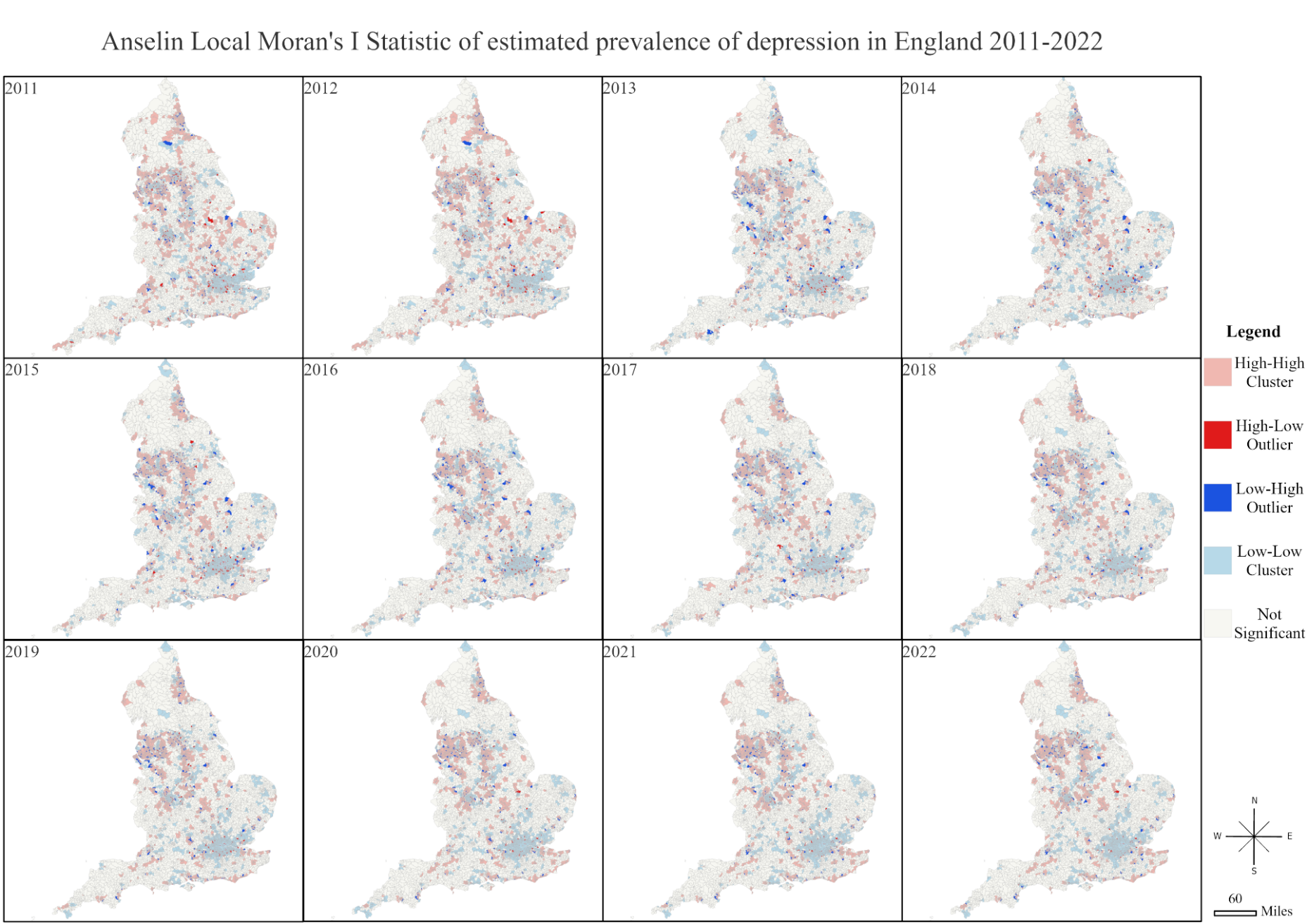


**Figure S1. Maps of England by Lower Super Output Areas (LSOAs), showing the results of Anselin Local Moran’s I algorithm for depression prevalence from 2011-2022**


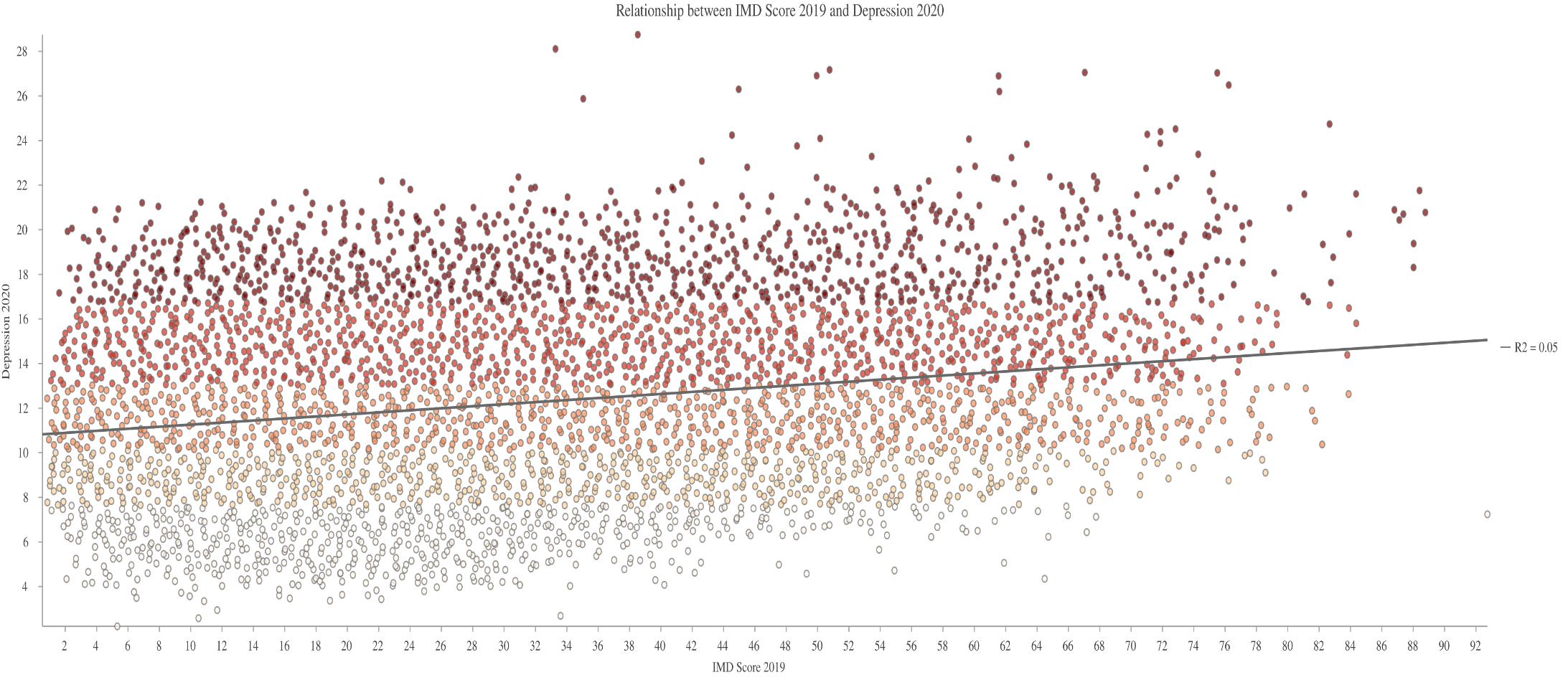


**Figure S2. Correlation coefficients scatter plot between Index of Multiple Deprivation (IMD) scores* in 2019 and Depression 2020 in all Lower Super Output Areas (LSOAs) in England (y = 10.8 + 0.046 x)**

*The exponential transformation for the production of IMD scores is described in detail in the following link:

<https://assets.publishing.service.gov.uk/government/uploads/system/uploads/attachment_data/file/833951/IoD2019_Technical_Report.pdf>
